# Systematic Investigation and Expression Profiles of the Nitrate Transporter 1/Peptide Transporter Family (NPF) in Tea Plant (*Camellia sinensis*)

**DOI:** 10.3390/ijms23126663

**Published:** 2022-06-15

**Authors:** Yongxin Wang, Kang Wei, Li Ruan, Peixian Bai, Liyun Wu, Liyuan Wang, Hao Cheng

**Affiliations:** Key Laboratory of Biology, Genetics and Breeding of Special Economic Animals and Plants, Ministry of Agriculture and Rural Affairs, National Center for Tea Improvement, Tea Research Institute Chinese Academy of Agricultural Sciences (TRICAAS), Hangzhou 310008, China; wyx@tricaas.com (Y.W.); weikang@tricaas.com (K.W.); ruanli@tricaas.com (L.R.); baipeixian2018@outlook.com (P.B.); wuly@tricaas.com (L.W.)

**Keywords:** *Camellia sinensis*, gene expression, nitrate, NPF

## Abstract

NRT1/PTR FAMILY (NPF) genes are characterized as nitrate and peptide transporters that played important roles in various substrates transport in plants. However, little is known about the NPF gene in tea plants. Here, a total of 109 CsNPF members were identified from the tea plant genome, and divided into 8 groups according to their sequence characteristics and phylogenetic relationship. Gene structure and conserved motif analysis supported the evolutionary conservation of *CsNPFs*. Many hormone and stress response *cis*-acting elements and transcription factor binding sites were found in *CsNPF* promoters. Syntenic analysis suggested that multiple duplication types contributed to the expansion of NPF gene family in tea plants. Selection pressure analysis showed that *CsNPF* genes experienced strong purifying selective during the evolution process. The distribution of NPF family genes revealed that 8 NPF subfamilies were formed before the divergence of eudicots and monocots. Transcriptome analysis showed that *CsNPFs* were expressed differently in different tissues of the tea plant. The expression of 20 *CsNPF* genes at different nitrate concentrations was analyzed, and most of those genes responded to nitrate resupply. Subcellular localization showed that both CsNPF2.3 and CsNPF6.1 were localized in the plasma membrane, which was consistent with the characteristics of transmembrane proteins involved in NO_3_^-^ transport. This study provides a theoretical basis for further investigating the evolution and function of *NPF* genes.

## 1. Introduction

Nitrogen (N) is an essential nutrient for plant growth and development. Nitrate is a major N source for higher plants and also serves as a signal to regulate plant development [1,2,3]. The NO_3_^−^ uptake system consists of low-affinity transport system (LATS) and high-affinity transport systems (HATS), which are mainly mediated by nitrate transporter 1/peptide transporter (NRT1/PTR) and nitrate transporter 2 (NRT2), respectively [4,5]. NRT1 belongs to the large PTR family, in which family members are also described as proton-coupled oligopeptide transporter (POT) or solute carrier 15 (SLC15) [5,6,7]. A study involving 31 fully sequenced plant genomes unified these proteins under the name NPF (NRT1/PTR FAMILY) [7]. 

The first functionally characterized NPF protein, AtNPF6.3/AtNRT1.1/CHL1, was identified in *Arabidopsis thaliana* as a nitrate transporter [8]. Since then, its homologs have been cloned and functionally characterized in various plants [9,10]. The NPF proteins well known for their roles in nitrate transport, are considered to be the main components of LATS of high NO_3_^−^. However, some NPF proteins, such as AtNPF6.3 in *A. thaliana* and MtNRT1.3 in *Medicago truncatula*, were dual-affinity transporters participating in both HATS and LATS [11,12]. These NPF proteins have a wide absorption range for both high and low nitrate concentrations. ZmNPF6.6 in *Zea mays* and MtNIP/LATD in *M. truncatula* have been shown to be high-affinity nitrate transporters [13,14]. To date, at least 21 *Arabidopsis* NPF members have been shown to transport nitrate, and many of them are also able to transport other substrates [15,16]. 

Tea plant, *Camellia sinensis* (L.) O. Kuntze, is an economically important crop and its leaves are the raw material for producing the non-alcoholic beverage “tea”. Tea is rich in tea polyphenols, theanine and other active substances beneficial to human health [17,18]. To improve the quality of tea, a large amount of nitrogen fertilizer was applied to increase the content of free amino acids in tea leaves. The N input in tea garden is about 2–5 times that of other crops [19]. Therefore, it is particularly important to understand the molecular mechanism of nitrogen uptake in tea plants. To date, NPF family genes have been identified in several species, such as *Arabidopsis*, rice [20], poplar [21], apple [22], sugarcane [23], wheat [16] and spinach [24]. However, research on NPF family gene in tea plants is still limited. Due to its important function, it is necessary to make further in-depth investigations of *NPF* genes in tea plants. 

In this study, a total of 109 *CsNPF* genes were identified in the tea plant genome. The phylogenic distribution, chromosomal location, gene structure, conserved motifs, duplication pattern, and selection pressure of *NPF* genes in tea plant were systematically analyzed. Furthermore, we performed expression profile analysis of NPF genes in different tissues of tea plant. The expression of *CsNPF* genes under different nitrate concentrations was detected by reverse transcription-quantitative real-time PCR (RT-qPCR). This study provides a basis for studying the evolution and function of *NPF* genes in tea plant.

## 2. Results 

### 2.1. Identification of Tea Plant CsNPFs 

A total of 109 CsNPF protein sequences were identified from the tea plant genome (Tables S1 and S2). To better distinguish these genes, we named *CsNPF* genes as *CsNPF1.X*~*CsNPF8.X* according to the previously reported rules [7]. The lengths of the CsNPF proteins ranged from 72 (CsNPF5.17) to 957 (CsNPF2.5) amino acids, with molecular weight ranging from 7.8 kD to 105.81 kD and theoretical p*I* values ranging from 4.43 (CsNPF2.9) to 9.98 (CsNPF5.17). Subcellular localization prediction indicated that most CsNPFs were localized to the plasma membrane (Table S1).

### 2.2. Phylogenetic Analysis of CsNPFs

**Figure 1:**
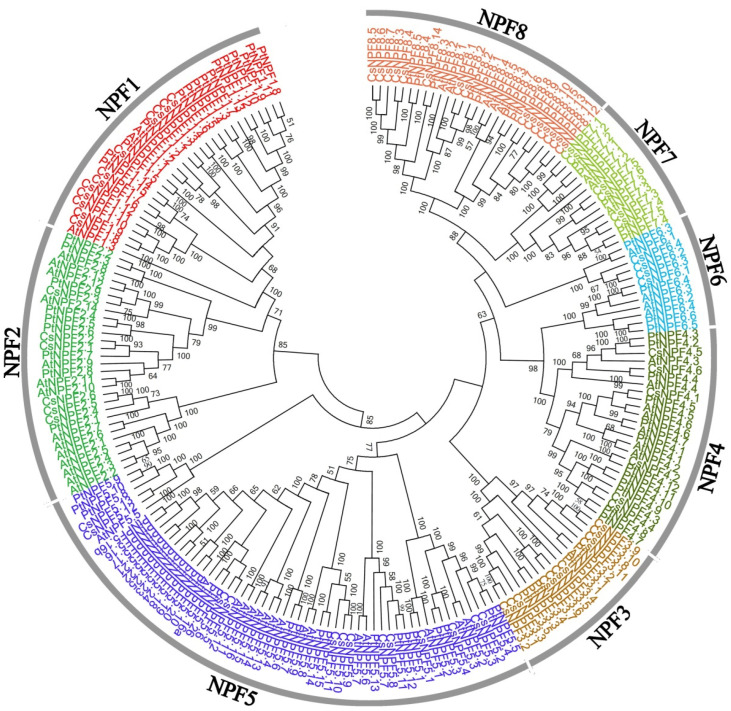
Phylogenetic tree analysis of NPF proteins from *C. sinensis*, *A. thaliana*, and *P. Trichocarpa*.

**Figure 2:**
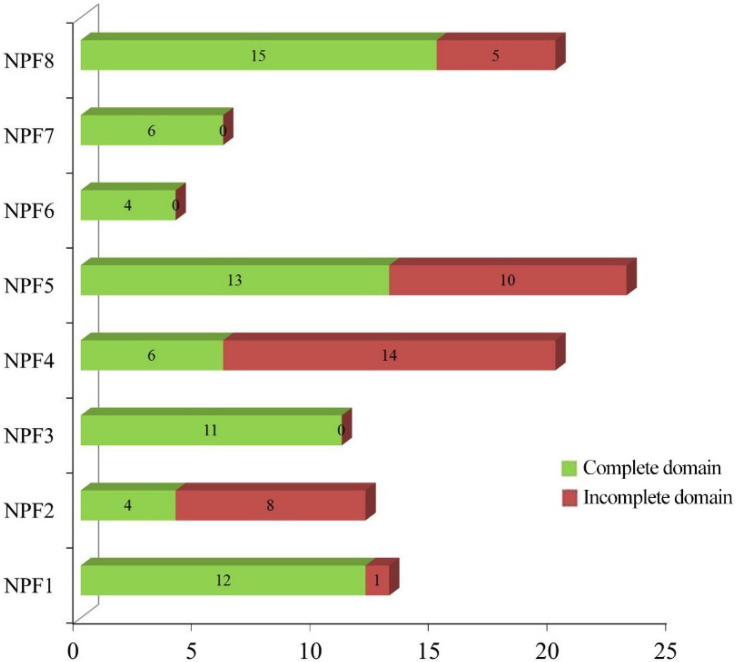
Distribution of CsNPF genes in different subfamilies.

To investigate the evolutionary relationship of *CsNPFs*, multi-sequence alignment of the 53 AtNPFs, 85 PtNPFs, and 109 CsNPFs was conducted by using the clustalx1.83 software and the result was used for the construction of an unrooted phylogenetic tree. Notably, 36 CsNPFs were removed from the phylogenetic tree construction due to C-terminal or N-terminal deletions. According to the phylogenetic tree (Figure 1), the *CsNPFs* were divided into 8 subfamilies (NPF1–NPF8). Among the 8 subfamilies, the NPF5 subfamily (23) contained the most *NPF* genes, while the NPF6 subfamily (6) contained the least (Figure 2 and Table S2). All members of the NPF3, NPF6, and NPF7 subfamilies contained complete domains, while 70% of the members of the NPF4 subfamily contained incomplete domains. 

### 2.3. Distribution of NPF Members in Different Plant Species

**Figure 3:**
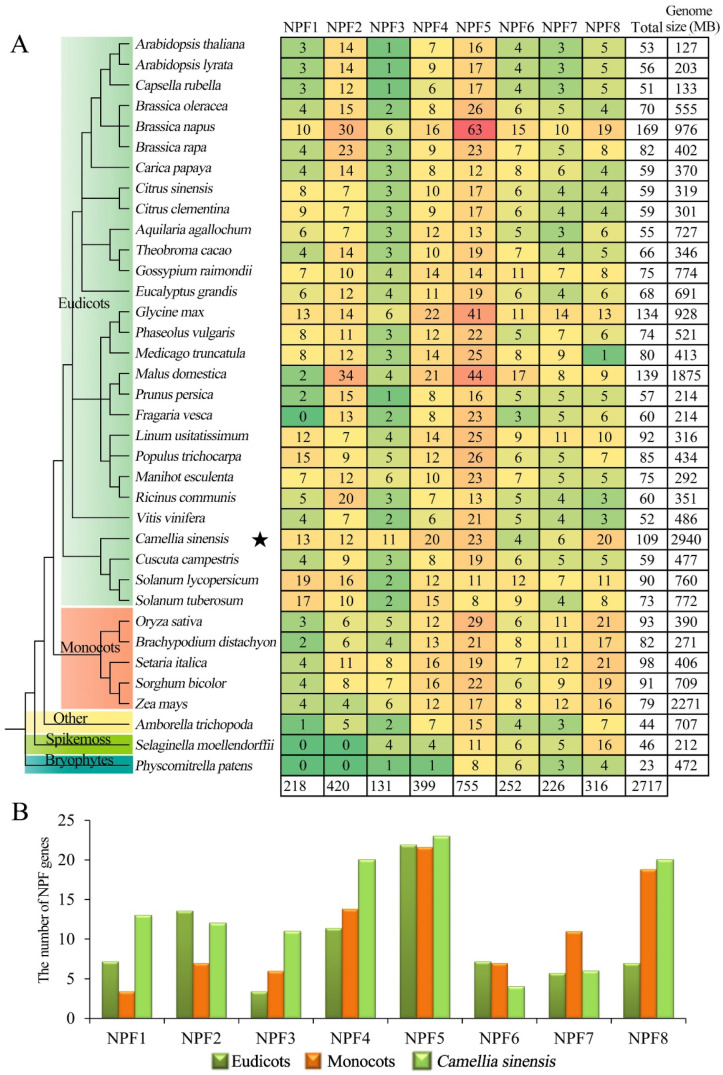
Comparison of NPF genes in different species. (**A**) Classification of NPF genes from 36 plant species. (**B**) Distribution of NPF genes in tea plant compared with the average number in eudicots and monocots. The phylogenetic tree of the 36 plant species was downloaded from the NCBI website (https://www.ncbi.nlm.nih.gov/Taxonomy/CommonTree/wwwcmt.cgi (accessed on 15 October 2021)) and reconstructed by MEGA5.

To investigate the evolution of CsNPF in plant species, a comparison of NPF members in different plant species was conducted. Information on NPF family members in other species was extracted from previous studies [7,25]. A total of 36 plant species were analyzed, including 28 eudicots, 5 monocots, 1 other angiosperm (*Amborella trichopoda*), 1 spike moss (*Selaginella moellendorffii*), and 1 bryophyte (*Physcomitrella patens*). As shown in Figure 3, eight NPF subfamily members are present in almost all species, while NPF1 and NPF2 subfamily members are absent in lower plants (*S. moellendorffii* and *P. patens*). The NPF5 subfamily has the largest number of members of almost all species, especially in *B. napus*, with 63 members. The distribution of NPF members in eudicots and monocots was significantly different in different NPF subfamilies. The NPF1 and NPF2 subfamilies members were more abundant in eudicots than in monocots, while more NPF3, NPF7 and NPF8 subfamilies members were found in monocots. In the NPF6 subfamily, the number of NPF members in tea plant was less than the average of eudicots and monocots. However, in NPF1, NPF3, NPF4 and NPF8 subfamilies, the number of NPF members in tea plant was much more than the average number of eudicots, which was 1.8, 3.3, 1.8, and 2.9 times the number of of eudicots, respectively. 

### 2.4. Gene Structure and Motif Analysis of CsNPFs

**Figure 4:**
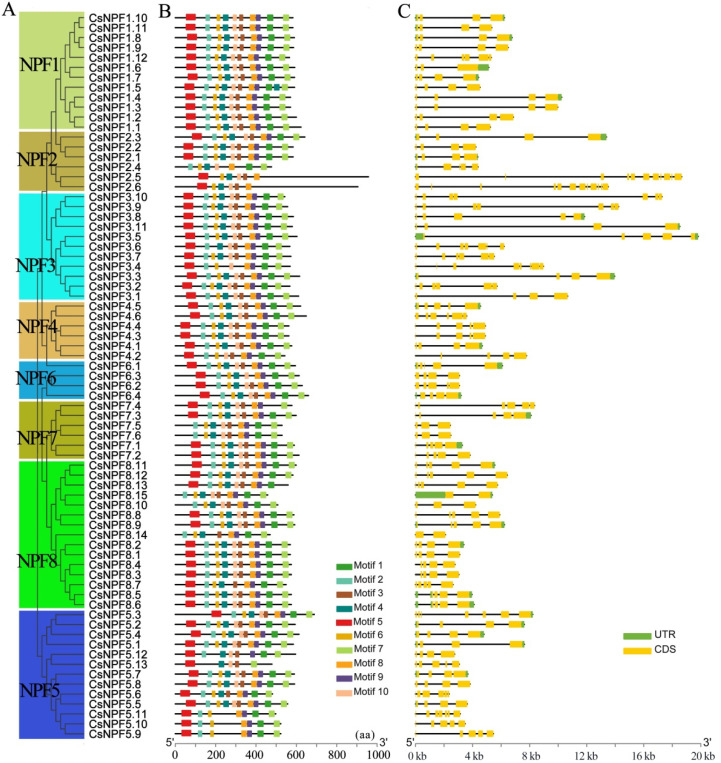
Gene structure and conserved motif compositions of *CsNPF* genes. (**A**) Phylogenetic tree of CsNPFs. (**B**) Conserved motifs of CsNPF proteins. The motifs 1–10 are displayed in different colored boxes. (**C**) Gene structure of *CsNPF* genes. The green and yellow squares represent the untranslated region (UTR) and coding sequences (CDS), respectively.

To characterize the protein sequences of the 73 CsNPF family members, 10 distinct motifs were predicted by the MEME program (Figure 4A). About 71% of CsNPF proteins contained all 10 conserved motifs, while other proteins appeared to be missing one or more of these motifs. Among them, all members of the NPF1 subfamily contain 10 motifs. Most closely related members share common motif compositions and arrangement orders. For example, motifs 1, 7, and 9 were lost in CsNPF2.5, and CsNPF2.6 of NPF2 subfamily. Motifs 3, 4 and 10 were absent in CsNPF5.9, CsNPF5.10 and CsNPF5.11 of NPF5 subfamily. 

Gene structure is an important parameter for gene evolution that further supports phylogenetic trees [26]. The coding sequences (CDS) of *CsNPF* genes were further analyzed to determine the structural diversity of these genes (Figure 4B). The numbers of introns of *CsNPF* genes ranged from 1 to 11. Of the 73 *CsNPF* genes, 53 (73%) had 3 or 4 introns. Fourteen *CsNPF* genes contained more than 4 introns, and 6 of them belonged to the NPF3 subfamily. *CsNPF2.5* and *CsNPF2.6* have the largest number of introns, with 10 and 11 introns respectively. However, both *CsNPF8.14* and *CsNPF8.15* contain only one intron. Overall, the most closely related *CsNPF* genes shared similar gene structures in terms of intron number and exon length. 

### 2.5. Chromosome Distribution of CsNPFs

**Figure 5:**
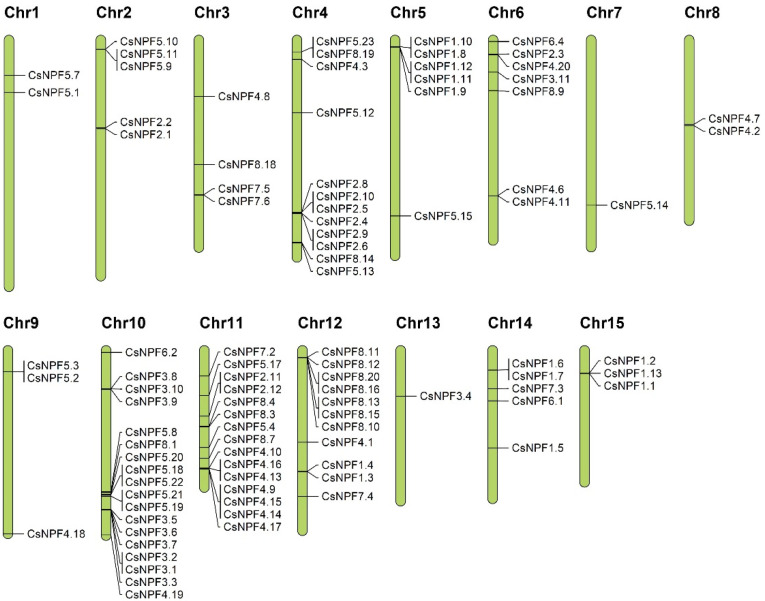
Chromosomal locations of *CsNPF* genes in tea plant genome.

The chromosomal location of each *CsNPF* gene was analyzed based on tea plant genomic annotation information. As shown in Figure 5 and Table S1, 95 out of the 109 *CsNPF* were mapped on the 15 chromosomes, while the remaining 14 genes were located on Contig. The distribution of *CsNPF* on each chromosome was extremely uneven. For example, Chr10 possesses the most *CsNPF* genes (18), followed by Chr11 (15 genes), while Chr7 and Chr13 contain only one *CsNPF* gene. Notably, NPF3 subfamily genes have an obvious preference for Chr10, with 9 out of 10 *CsNPF* located on Chr10.

### 2.6. Expansion and Evolution of CsNPF Genes

**Figure 6:**
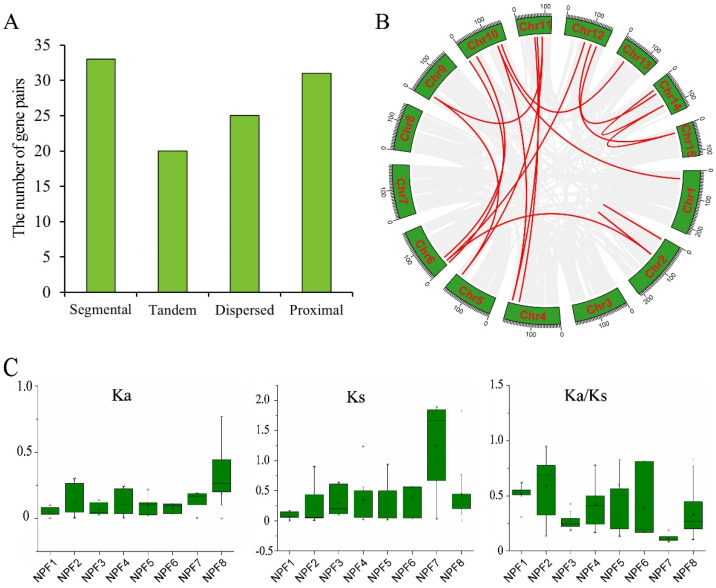
The expansion and evolution analysis of *NPF* genes in tea plant. (**A**) The number of duplication types of *CsNPF* genes. (**B**) Syntenic relationships of *CsNPF* genes on tea plant chromosomes. (**C**) Values of Ka, Ks, and Ka/ Ks for *CsNPF* gene pairs of 8 subfamilies. Gray lines in the background indicate syntenic blocks in the whole tea plant genome, and red lines indicate segmental duplicates of *CsNPF* gene pairs.

To study the evolutionary processes of CsNPF family, the gene duplication pattern of *CsNPF* genes was analyzed (Figure 6 and Table S1). Thirty-three of the 109 *CsNPF* genes were found to be segmentally duplicated, 31 of which were located on chromosomes 1, 2, 4, 5, 6, 9, 10, 11, 12, 13, 14, and 15, and 2 on Contig (Figure 6A). There were 21 pairs of syntenic relationships in tea plant (Figure 6B). A total of 13 tandem repeats containing 20 *CsNPF* genes were identified. In addition, 25 and 31 *CsNPFs* were also found to be dispersed and proximal duplicates, respectively, which may also contribute to the expansion of the *CsNPF* family genes.

To further study the selection pressure of tea plant *NPF* genes during evolution, the values of synonymous (Ks) and nonsynonymous (Ka) substitution rates, and Ka/Ks ratios for each duplicated pair from different subfamilies were calculated (Figure 6C and Table S3). The mean values of Ka of gene pairs in NPF1-NPF8 subfamilies were 0.05, 0.12, 0.07, 0.12, 0.10, 0.08, 0.14, and 0.09, respectively. The mean values of Ks were 0.09, 0.27, 0.29, 0.34, 0.35, 0.39, 1.24, and 0.44, respectively. The mean values of Ka/Ks were 0.52, 0.59, 0.27, 0.42, 0.6, 0.39, 0.11, and 0.33, respectively. Among the results, the Ks of the NPF7 subfamily was much higher than that of other NPF subfamilies, suggesting that they may have originated from more ancient duplication events. Except for *CsNPF5.18*/*CsNPF5.19*, the Ka/Ks ratio of almost all NPF duplicated pairs was less than 1, especially in the NPF7 subfamily. The results indicate that tea plant *NPF* genes might suffer from strong purifying selection throughout the long evolutionary events.

### 2.7. Synteny Analyses of NPF Genes between Tea Plant and Six Representative Species

**Figure 7:**
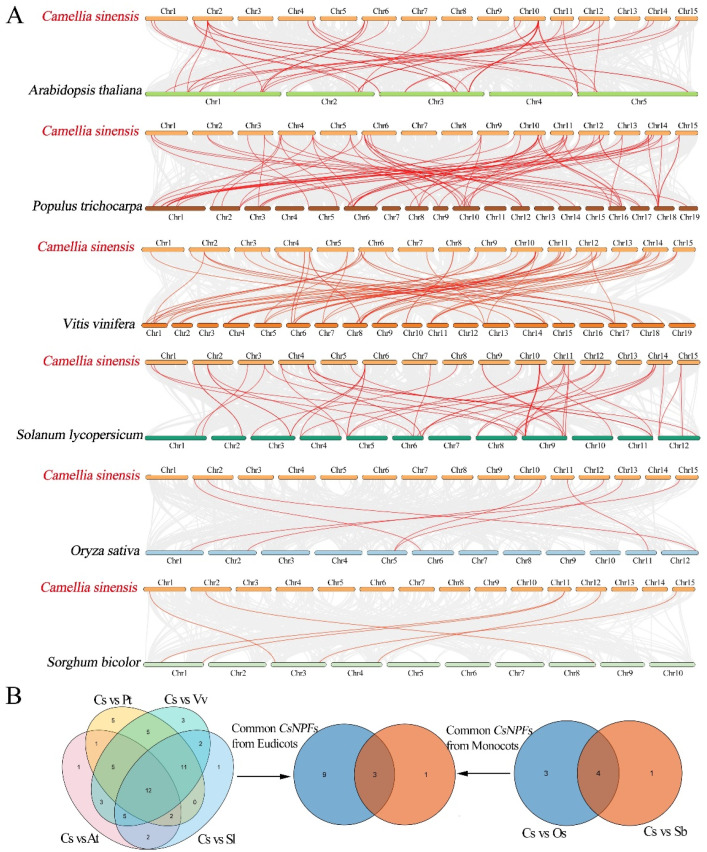
Synteny analyses of the *NPF* genes between *C. sinensis* (Cs) and six representative plant species. (**A**) Synteny analyses of the *NPF* genes between *C. sinensis* and other six plant species. (**B**) Venn diagram analysis of collinear *NPF* genes between *C. sinensis* and other six plant species. Four eudicots include *A. thaliana* (At), *P. trichocarpa* (Pt), *V. vinifera* (Vv) and *S. lycopersicum* (Sl); 2 monocots include *O. sativa* (Os) and *S. bicolor* (Sb).

To further explore the potential evolutionary mechanisms of the tea plant NPF family, we selected 6 representative species, including 4 eudicots (*A. thaliana*, *P. trichocarpa*, *Vitis vinifera*, and *Solanum lycopersicu*) and 2 monocots (*O. sativa*, and *Sorghum bicolor*), to construct comparative syntenic maps with tea plant (Figure 7 and Table S4). The results showed that the tea plant has more gene pairs with eudicots than with monocots. There were 75 pairs of syntenic pairs between tea plant and *P. trichocarpa*, followed by *V. vinifera* (65), *S. lycopersicu* (52), *A. thaliana* (45). However, there were only 7 and 6 pairs of syntenic relationships of tea plant with *O. sativa*, and *S. bicolor*, respectively. Among them, 3 NPF genes, *CsNPF1.2*, *CsNPF2.2*, and *CsNPF7.4*, were simultaneously identified between tea plant and the other six species, indicating that these genes may have vital roles in the evolution of the NPF gene family.

### 2.8. Regulatory Mechanism in the Promoter Regions of CsNPF Genes

**Figure 8:**
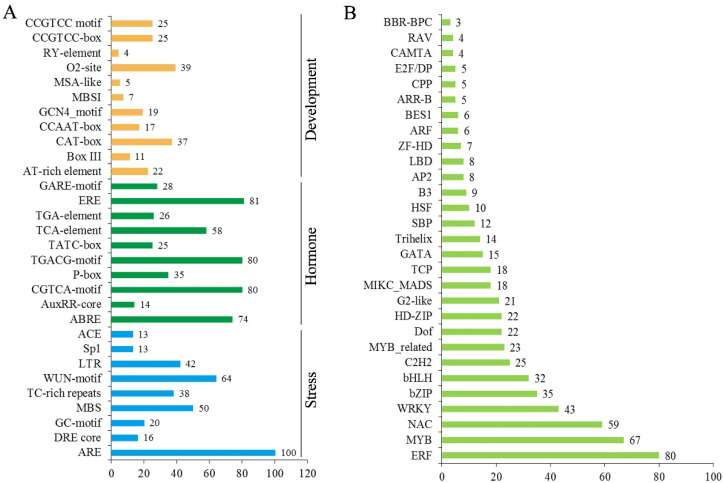
Analysis of *cis*-acting regulatory elements and TF binding site in the *CsNPF* promoters. (**A**) Putative *cis*-acting regulatory elements of *CsNPF* genes. (**B**) The top 30 riched TF gene families with potential binding sites in the *CsNPF* promoter regions.

To understand the potential functions of *CsNPF* genes, the *cis*-acting regulatory elements (*CREs*) in promoter regions of *CsNPFs* were predicted using PlantCARE. A total of 111 types of *CREs* were identified from 109 *CsNPF* gene promoters (Table S5). Each *CsNPF* contained 20 to 40 *CREs* in the promoter region, and some closely related genes showed similar *CREs* type. Several common *CREs*, such as CAAT-box and TATA-box and some light-responsive elements (G-box, Box 4, GT1-motif and TCT-motif) were obtained. Meanwhile, some *CREs* were shown to respond to stresses, such as drought-inducible MYB binding site elements (MBS, 50), low temperature-responsive elements (LTR, 67), wound-responsive elements (WUN-motif, 64), and anaerobic-inducible element (ARE, 100) (Figure 8A). Large numbers of *CREs* were involved in hormone responses, such as MeJA-responsive elements (TGACG-motif and CGTCA-motif), gibberellin-responsive elements (TATC-box, P-box and GARE-motif), Auxin (TGA-element and AuxRR-core), abscisic acid-responsive elements (ABREs), Ethylene (ERE) and salicylic acid reaction (TCA-elements) elements. In addition, many transcription factor (TF) binding sites were identified, such as WRKY binding sites (W box) and MYB binding sites (MRE, MYB, MBS, and MBSII).

Transcription factors regulate the expression of target genes by binding to *CREs* in promoters. To further explore the transcriptional regulation of *CsNPFs*, we speculated the potential TFs that bind to the *CsNPFs* promoters (Figure 8B and Table S6). The results showed that 612 TF genes from 43 TF gene families had potential target binding sites in the CsNPFs promoter regions. Among the 612 TFs, MYB (90) gene family was the most abundant, followed by ERF (80), NAC (59), WRKY (43), bZIP (35), bHLH (32), and C2H2 (25) gene family.

### 2.9. Expression Patterns of CsNPFs in Different Tissues

**Figure 9:**
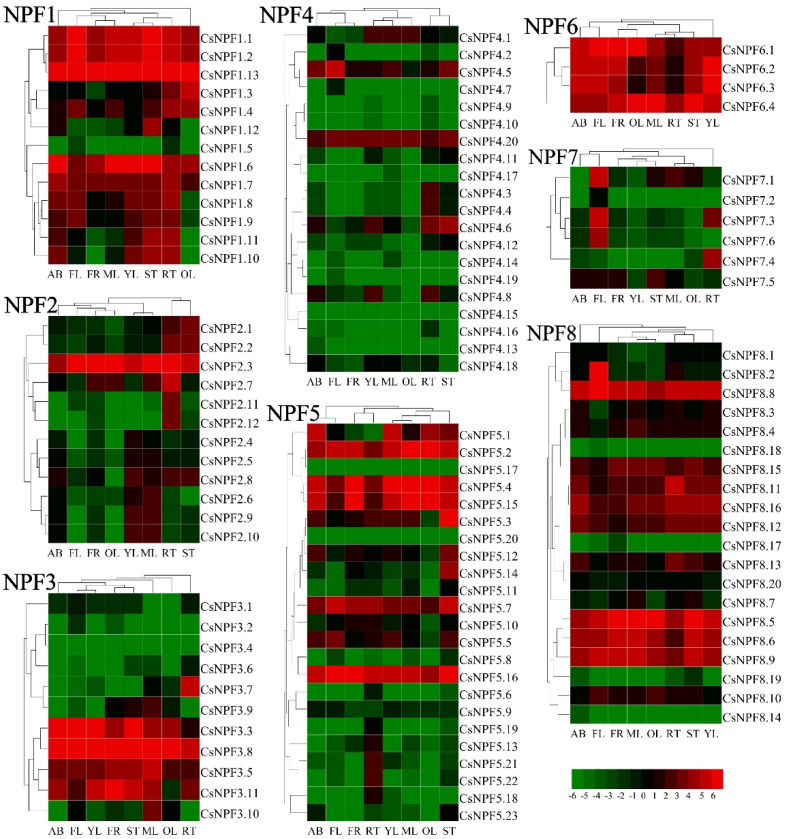
Expression heatmap of *CsNPF* genes in 8 tissues of tea plant. The tissues are apical buds (AB), young leaves (YL), mature leaves (ML), old leaves (OL), young stems (ST), flowers (FL), young fruits (FR), and tender roots (RT). Gene expression level was evaluated log2 transformed RPKM value. Red represents high expression. Green indicates low expression.

To investigate the expression patterns of NPF genes in tea plant, we analyzed the expression levels of *CsNPFs* in 8 tissues (apical buds, young leaves, mature leaves, old leaves, immature stems, flowers, young fruits, and tender roots) (Figure 9 and Table S7). Of 109 *CsNPF* genes, 60 (55%) were ubiquitously expressed in all 8 tissues; 39 (36%) were low expressed with meanFPKM < 1. *CsNPF* genes showed different expression characteristics in different subfamilies. In NPF1, NPF6, and NPF8 subfamilies, most *CsNPF* genes were expressed at high levels in most tissues. In particular, all members of NPF6 subfamily genes were highly expressed in almost all tissues. Most members of the NPF4 and NPF7 subfamily genes showed lower expression levels. Some *CsNPF* genes displayed tissue-specific or preferential expression patterns. For example, in NPF3 subfamily, *CsNPF2.6*, *CsNPF2.9* and *CsNPF2.10* were preferentially expressed in young and mature leaves, while *CsNPF2.7*, *CsNPF2.11* and *CsNPF2.12* were preferentially expressed in root. In the NPF2 subfamily, *CsNPF3.7* was preferentially expressed in root, while *CsNPF3.10* was preferentially expressed in mature leaves. *CsNPF5.13*, *CsNPF5.18*, *CsNPF5.21* and *CsNPF5.22* in NPF4 subfamily were mainly expressed in root. In the NPF7 subfamily, *CsNPF7.1*, *CsNPF7.3* and *CsNPF7.6* had higher expression levels in flowers, while *CsNPF7.3* and *CsNPF7.4* were preferentially expressed in roots. 

### 2.10. Expression of CsNPFs under Different Nitrate Concentrations

**Figure 10:**
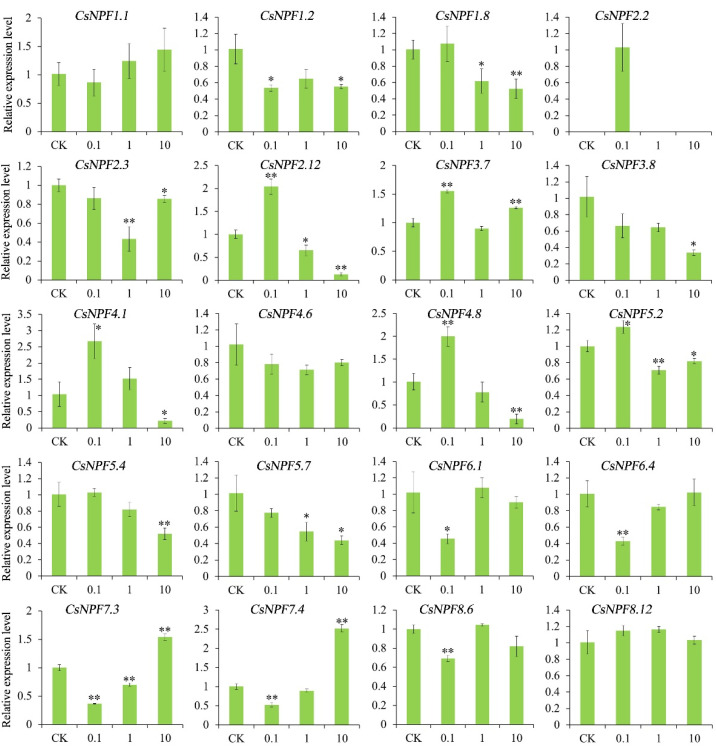
Expression of selected *CsNPF* genes under different nitrate concentrations. Error bars represent standard deviation (SD). “*” and “**” represent the significance level of 0.05 and 0.01, respectively.

Nitrate is the main substrate for NPF protein transport. Here, the expression profiles of 20 *CsNPF* genes were detected under different nitrate concentrations. The 20 *CsNPF* genes were selected from the 8 subfamilies, which were representative and can explain the expression profiles of genes from different subfamilies (Figure 10). *CsNPF1.2*, *CsNPF1.8*, *CsNPF3.8*, *CsNPF5.4*, and *CsNPF5.7* were down-regulated with the increase of nitrate concentration. *CsNPF2.2* was detected only at 0.1 mM nitrate concentration. *CsNPF6.1* and *CsNPF6.4* were decreased under 0.1 mM nitrate concentration. *CsNPF2.12*, *CsNPF4.1* and *CsNPF4.8* were induced under low nitrogen and then decreased with increasing nitrogen concentration. *CsNPF7.3* and *CsNPF7.4* showed an opposite trend, decreasing first and then increasing. 

### 2.11. Subcellular Localization of CsNPF2.3 and CsNPF6.1

**Figure 11:**
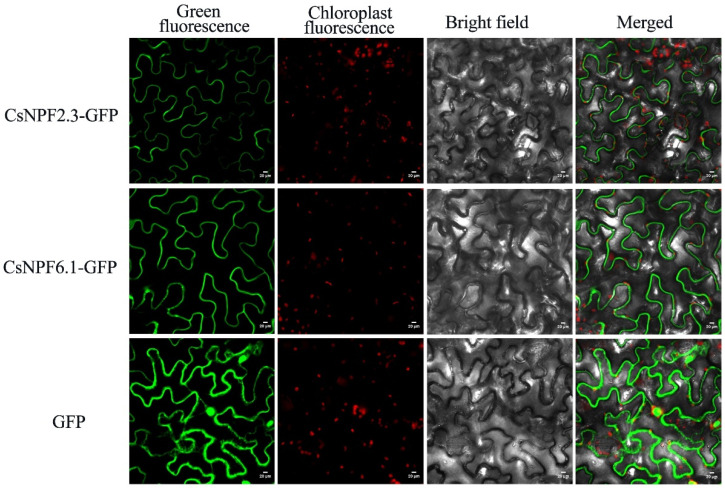
Subcellular localization of CsNPF2.3 and CsNPF6.1 proteins.

The *CsNPF2.3-GFP* fusion gene, *CsNPF6.1-GFP* fusion gene and empty vector containing *GFP* were transformed into tobacco epidermal cells. The results showed that the fusion proteins CsNPF2.3 and CsNPF6.1 were specifically located in the plasma membrane of tobacco leaves cells, and control GFP signals were observed in the nucleus, cytoplasm, and cell membrane (Figure 11). These results indicated that CsNPF2.3 and CsNPF6.1 were located in the plasma membrane, which was consistent with the results predicted by bioinformatics and consistent with the characteristics of transmembrane proteins involved in NO_3_^−^ transport.

## 3. Discussion

NPF genes comprise a large family of members widely distributed in eukaryotes. An increasing number of studies has confirmed that NPF genes are involved in the transport of nitrate/nitrite [8], di/tri-peptides [27], and various hormones [28,29,30]. As an important cash crop, tea plant is widely planted in China and all over the world because of its functional activity and important economic value. Tea plant is a plant that needs a lot of nitrogen fertilizer [19]. So far, functional studies of NPF gene have been carried out in Arabidopsis, rice, and other plants, but almost no reports on tea plant. In this study, 109 *CsNPF* genes were identified from the tea plant genome and divided into 8 subgroups based on the similarity of structure. 

It is widely believed that the variation in gene structure is one of the representative traces of gene family evolution. According to the analysis of gene structure and conserved motifs, CsNPF was relatively conserved structurally. Most CsNPFs contain all 10 motifs, and no specific motifs belonging to a subfamily have been found. In the same subfamily, some closely related genes show the same motif deletion. Analysis of gene structure showed that most *CsNPF* genes (73%) contained 3 or 4 introns. Similar results have been reported in *Arabidopsis*, *P. trichocarpa*, soybean [31], and *B. napus* [25]. On the whole, *CsNPFs* within the same subfamily shared similar gene structures and motifs. Changes in gene structure and conserved motifs may be important reasons for the functional diversification of tea plant NPF family genes.

Gene duplication and loss events are the main drivers of species evolution, promoting the expansion of gene families and the generation of novel genes [32]. NPF genes are widely distributed in all subfamilies of 36 species, although NPF1 and NPF2 subfamilies are absent in lower plants *S. Moellendorffii* and *P. Patens*. In original angiosperms, all 8 subfamilies were present, suggesting that the duplication and diversification of NPF genes may have occurred before the differentiation of eudicots and monocots, and that NPF gene duplication in eudicots and monocots was an independent process. More NPF1 and NPF2 subfamily members were found in eudicots, while more NPF3, NPF7 and NPF8 subfamily members were found in monocots. The number of NPF1, NPF3, NPF4 and NPF8 subfamily members in tea plant was higher than that in eudicots, indicating these subfamilies seem to be more active in duplication and may have more function in the tea plant.

Gene family expansion mainly depends on tandem, segmental, or whole-genome duplications [33,34]. Recently completed genome sequencing of the tea plant indicated that tea plant has undergone two rounds of whole-genome duplications (WGD) [35,36,37]. Moreover, the large number of genes in the *CsNPF* family suggests that it evolved through many duplication events in tea plant. In *B. napus*, 137 (71%) of 193 *BnaNPFs* were found to be segmentally duplicated, and only 2 genes were identified as tandem duplication, suggesting that segmentation duplication was the main driver of NPF gene family expansion in *B. napus* [34]. Here, 33 (30%) and 20 (18%) of 109 *CsNPFs* were identified involved in segmental duplication and tandem events, respectively. Dispersed and proximal duplicates were also identified in the tea plant NPF gene family. These results indicated that the expansion forms of the NPF gene family in tea plant were more diversified. The number of NPF3 subfamily in tea plant was 3.3 times that in eudicots. Notably, there are two segmental and two tandem duplications in the NPF3 subfamily of tea plant, which may account for the higher number of NPF3 subfamily genes in tea plant than in other species. Few gene pairs were identified between tea plant and two monocots; while numerous colinearity gene pairs were found between tea plant and other 4 eudicots because of their close relationship. The results showed that the NPF gene differentiated rapidly in monocots and eudicots. In addition, three *CsNPF* genes, *CsNPF1.2*, *CsNPF2.2*, and *CsNPF7.4*, were colinear paired with all six species, suggesting that these colinear pairs already existed before the differentiation of monocots and eudicots ancestors. 

The expression pattern of a gene is usually closely related to its *CREs* [38]. Many *CREs* related to development, stress, and phytohormone were detected in the *CsNPF* promoter regions, suggesting that *CsNPFs* may play different roles in biological processes. Numerous studies have demonstrated that NPF can transport hormone substrates. In *Arabidopsis*, at least 9 NPF members have ABA transport function and 18 NPF members have GA transport function [28]. In *B. napus*, 32.66% of *BnaNPF* expressions (65/199 gene) were regulated by at least one hormone [39]. In this study, most *cis*-elements involved in phytohormone responses, such as MeJA- (80/109 genes), Ethylene- (81/109), and ABA-responsive *CRE* (74/109 genes), were found in a series of *CsNPF* promoters, suggesting their potential hormone-inducing characteristics. The absorption and transport of nitrogen plays an important role in plant stress adaptation [40,41]. AtNPF6.3/AtNRT1.1 is involved in regulating drought tolerance of *Arabidopsis* [42]. AtNRT1.5 plays a negative role in salt tolerance and drought resistance in *Arabidopsis*, regulating the long-distance transport and spatial distribution of Na^+^, and affecting the expression of stress-responsive genes [43]. Many stress-related elements related to low temperature (LTR), drought (MBS), anaerobic (ARE) and wound (WUN-Motif) were also identified in the *CsNPF* promoters. Previous studies have shown the transcription factor AtERF59 binds directly to the *AtNRT1.8* promoter and ethylene insensitive 3 (EIN3) binds to the *AtNRT1.5* promoter [44]. *Arabidopsis* AtMYB59 positively regulates *AtNRT1.5* expression to coordinate root-to-shoot K^+^/NO_3_^–^ transport [45]. Many transcription factor binding sites were found in the *CsNPF* promoter regions. The results suggested that the expression of *CsNPF* genes might be regulated by multiple transcription factors.

Genes expressed in given tissues may have specific tissue functions, which provides crucial clues to understanding gene functions [46]. The *CsNPF* genes showed complex expression patterns, and closely related genes in the same subfamily showed similar expression patterns, reflecting the consistency of structure and function. Some *CsNPF* genes showed obvious tissue preference. For example, *CsNPF2.6*, *CsNPF2.9*, *CsNPF2.10* were mainly expressed in leaf. *CsNPF4.5*, *CsNPF7.3*, *CsNPF7.6*, *CsNPF8.2* showed preferred expression in flower. Most of the tissue-specific expression genes are preferentially expressed in root, such as *CsNPF2.7*, *CsNPF2.11*, *CsNPF2.12, CsNPF3.7*, *CsNPF5.13*, *CsNPF5.21*, *CsNPF5.22*, *CsNPF7.3*, *CsNPF7.4*. Similarly, most rice tissue-specific genes were preferentially expressed in root [20]. *AtNPF6.3*/*AtNRT1.1* was a dual-affinity nitrate transporter mainly expressed in Arabidopsis root and involved in nascent organ development [47]. In *T. aestivum*, *TaNPF6.2* was predominantly expressed in root; *TaNPF6.1* was highly expressed in different tissues; while *TaNPF6.4* was preferentially expressed in spike and node, but low in root and leaf [16]. All 4 *CsNPF6* genes were highly expressed in tea plant except for the root and old leaf. The variation in expression patterns suggested the divergence of *AtNPF6.3* orthologues in different species. 

Nitrate is the main substrate for NPF protein transport [15,16]. In *Arabidopsis*, more than a third of the NPF genes have been shown to transport nitrates [16]. *AtNPF4.6*/*AtNRT1.2* has been reported to be expressed mainly in the root of Arabidopsis and is involved in constitutive nitrate uptake [48]. In rice, *OsNPF4.5* plays a key role in mycorrhizal NO_3_^−^ acquisition [49]. Here, the transcripts of *CsNPF4.1* and *CsNPF4.8* in tea plant were significantly up-regulated under low nitrate conditions while down-regulated under high nitrate conditions. AtNPF7.3/AtNRT1.5 has been reported to be involved in nitrate loading in root xylem and transport nitrate from root to shoot [50]. *OsNPF7.3* was mainly expressed in lateral root and stem of rice, and could improve nitrogen utilization efficiency in rice paddy [51]. Its homologous genes in tea plant were *CsNPF7.3* and *CsNPF7.4*, which were preferentially expressed in root, and first decreased and then increased with the increase of nitrate concentration. In this study, the expression of several *CsNPF* genes changed significantly, indicating that these genes may be involved in nitrate uptake.

## 4. Materials and Methods

### 4.1. Plant Materials and Treatments

Fourteen-month-old cutting seedlings from tea plant, Zhongming 6 hao, were selected for this study. As previously [52], tea seedlings were cultured in full nitrogen hydroponic solution for 2 months. Then the seedlings were transferred to the nitrogen-deficient (without N) nutrient solution for 2 weeks of N-starvation treatment. Afterward, the seedlings were cultivated in the nutrient solution containing 0.1, 1, and 10 mM nitrate, respectively. Nitrogen starvation was used as the control (CK). The white roots were harvested after 2 h of treatment, rapidly frozen in liquid nitrogen, and then stored at −80 °C.

### 4.2. Identification of Tea Plant NPF Genes

Fifty-three NPF sequences of *A. thaliana* were obtained from the TARI website (http://www.arabidopsis.org/ (accessed on 15 July 2021)). The tea plant genome sequences were downloaded from the Tea Plant Information Archive (TPIA) website (http://tpdb.shengxin.ren/ (accessed on 15 July 2021)) [36]. The putative CsNPFs of tea plant were obtained via screening tea plant genome sequences by BlastP search, using AtNPFs as queries. The candidate sequences were then submitted to Pfam (http://pfam.xfam.org/ (accessed on 15 July 2021)) and NCBI CD-search (https://www.ncbi.nlm.nih.gov/Structure/cdd/wrpsb.cgi (accessed on 15 July 2021)) to confirm the presence of the MFS_1 or PTR2 (PF00854) domain. TMHMM Server v. 2.0 (http://www.cbs.dtu.dk/services/TMHMM/ (accessed on 20 July 2021)) was applied for prediction of putative transmembrane (TM) regions of CsNPFs. Subcellular localization was predicted by the WoLF PSORT server (https://wolfpsort.hgc.jp/ (accessed on 20 July 2021)).

### 4.3. Phylogenetic Analysis and Structural Characterization

Sequence alignments of NPF proteins from tea plant, *A. thaliana*, and *P. trichocarpa* were performed clustalx1.83 software with default parameters. The unrooted phylogenetic tree was constructed using MEGA 5.0 software with the neighbor-joining (NJ) methods, bootstrapping with 1000 replicates. NPF sequences of *A. thaliana* and *P. trichocarpa* were obtained from previous reports (Table S8) [53]. The exon/intron structures of *CsNPF* genes were obtained from tea plant genome annotation [36]. Conserved motifs were analyzed using the Multiple Em for Motif Elicitation 5.4.1 (MEME, https://meme-suite.org/meme/ (accessed on 9 November 2021)) program. 

### 4.4. Cis-Element Analysis

The sequence of 2000 bp upstream of the *CsNPF* gene transcription start site (ATG) was considered as promoter sequence. These promoter sequences were used to evaluate the putative *cis*-acting regulatory elements (*CREs*) using the PlantCARE database (http://bioinformatics.psb.ugent.be/webtools/plantcare/html/ (accessed on 9 November 2021)). The transcription factor (TF) binding sites in *CsNPF* promoter sequences were predicted using the PlantTFDB database (http://planttfdb.gao-lab.org/ (accessed on 9 November 2021)).

### 4.5. Chromosomal Arrangement and Gene Duplication of CsNPF Genes

Physical positions of 109 *CsNPF* genes were extracted from the tea plant genome database. Mapchart software was used to visualize the chromosome localization [54]. Multiple Collinear Scan Toolkit (MCScanX) was used to determine the gene duplication events and collinearity relationships of tea plant *NPF* genes [55]. A chromosome region containing two or more homologous genes within a 200 kb range is defined as a tandem duplication event. The syntenic analysis maps of tea plant and other selected plants were constructed and visualized using the MCScanX and TBtools software [55,56]. The synonymous (Ks) and nonsynonymous (Ka) substitution rates, and Ka/Ks ratios for each duplicated pair (similarity of aligned regions > 70%) were calculated based on CDS alignments of the *CsNPF* genes using TBtools [56]. 

### 4.6. Transcript Abundance Analysis

For expression profiling of tea plant *NPF* genes, we utilized the transcriptome sequencing data of eight tissues of tea plant that were derived from the Tea Plant Information Archive (TPIA) (http://tpia.teaplant.org/ (accessed on 20 July 2021)) [35]. The eight tea plant tissues were apical buds (AB), young leaves (YL), mature leaves (ML), old leaves (OL), immature stems (ST), flowers (FL), young fruits (FR), and tender roots (RT). The expression cluster of *CsNPF* was analyzed and displayed by HemI 1.0 software.

### 4.7. RNA Isolation, Reverse Transcription, and RT-qPCR Detection

Total RNAs were extracted from samples using the EASYspin plant RNA extraction kit (Aidlab Biotechnologies, Beijing, China), and first-strand cDNA synthesis was performed using the PrimeScript RT reagent kit (TaKaRa, Dalian, China). RT-qPCR was performed using a LightCycler 480 machine (Roche Diagnostics) with SYBR Green reagents (Takara, Japan). RT-qPCR was performed in a 10 μL reaction mixture consisting of 5 µL of SYBR Premix Ex, 1 µL of diluted template cDNA, 0.3 µL of each primer, and 3.4 μL of ddH_2_O. The PCR amplification profile was as follows: denaturation at 95 °C for 30 s, followed by 40 cycles of 95 °C for 10 s, 58 °C for 10 s and 72 °C for 15 s. Three independent PCR reactions were performed on each gene, and the relative gene expression was calculated using the 2^−∆∆CT^ method [57]. Tea plant *GAPDH* was selected as the reference gene [52]. All primers used are listed in Table S9. 

### 4.8. Subcellular Localization

Subcellular localization analysis of tobacco epidermis was according to Zhang et al.’s method [58]. The *CsNPF2.3* and *CsNPF6.1* genes of the deletion termination codon were amplified using specific primers (Table S9) and inserted into the pBWA(V)HS-GFP vector. The constructed vector plasmid was transformed into *Agrobacterium* GV3101. The *Agrobacterium* cells were injected into the lower epidermis of one-month-old tobacco leaves and cultured under low light for 2 days. The GFP signals were observed and imaged under the confocal laser microscope (Zeiss, Germany).

## 5. Conclusions

This study performed a comprehensive analysis of *NPF* genes in tea plant. A total of 109 *CsNPF* genes were identified from the tea plant genome, and their gene structure, conserved motifs, chromosome location, *cis*-elements, gene duplication, evolutionary relationships, and expression patterns were systematically analyzed. Gene structure and motif analysis indicated that the CsNPF proteins were highly conserved in each subfamily. Syntenic analysis revealed that the expansion of tea plant NPF gene family was caused by multiple duplication types. The *CsNPF* family genes of tea plant experienced strong purifying selective pressure during evolution. Most of the selected *CsNPF* genes were induced by nitrate treatments. In addition, we proved that CsNPF2.3 and CsNPF6.1 were localized in the plasma membrane, which was consistent with the characteristics of transmembrane proteins involved in NO_3_^−^ transport. These results provide a valuable basis for studying the evolution and function of *CsNPF* genes, and aid in improving N absorption in tea plant breeding.

## Data Availability

Not applicable.

## Supplementary Materials

The following supporting information can be downloaded at: https://www.mdpi.com/article/10.3390/ijms23126663/s1.
